# 2-(1,2-Dimethyl-1*H*-indol-3-yl)-1-{5-[3-(1,3-dioxolan-2-yl)phen­yl]-2-methyl­thio­phen-3-yl}-3,3,4,4,5,5-hexa­fluoro­cyclo­pent-1-ene

**DOI:** 10.1107/S1600536811043509

**Published:** 2011-10-29

**Authors:** Li-qin Wang, Liu-shui Yan, Gang Liu

**Affiliations:** a College of Environmental and Chemical Engineering, Applied Chemistry, Nanchang Hangkong University, Nanchang 330034, People’s Republic of China; b Jiangxi Key Laboratory of Organic Chemistry, Jiangxi Science and Technology Normal University, Nanchang 330013, People’s Republic of China

## Abstract

The title compound, C_29_H_23_F_6_NO_2_S, a member of a new family of photochromic diaryl­ethene compounds having an unsymmetrically substituted hexa­fluoro­cyclo­pentene unit, displays dihedral angles between the indole and thio­phene rings of 52.5 (4)°, and between the indole ring and the planar C—C=C—C unit of the cyclopentene ring of 53.8 (6)°. The distance between the potentially reactive C atoms from the two heteroaryl substituents of 3.817 (6) Å is proven to be short enough for photocyclization to occur.

## Related literature

For literature on photochromic diaryl­ethene compounds, see: Pu *et al.* (2007 ▶, 2010 ▶); Yamamoto *et al.* (2003 ▶).
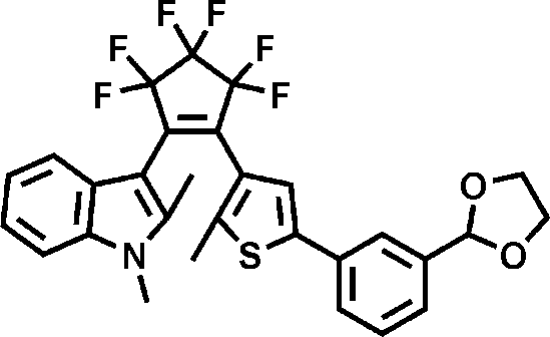


## Experimental

### 

#### Crystal data

C_29_H_23_F_6_NO_2_S
*M* *_r_* = 563.54
Monoclinic, 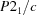

*a* = 10.7364 (13) Å
*b* = 9.8983 (12) Å
*c* = 24.020 (3) Å
β = 93.151 (1)°
*V* = 2548.8 (5) Å^3^
*Z* = 4
Mo *K*α radiation
μ = 0.20 mm^−1^
*T* = 296 K
0.50 × 0.38 × 0.30 mm

#### Data collection

Bruker SMART APEX diffractometer
Absorption correction: multi-scan (*SADABS*; Sheldrick, 1996 ▶) *T*_min_ = 0.906, *T*_max_ = 0.942
22213 measured reflections
5816 independent reflections
4512 reflections with *I* > 2σ(*I*)
*R*_int_ = 0.024

#### Refinement

*R*[*F* ^2^ > 2σ(*F* ^2^)] = 0.041
*wR*(*F* ^2^) = 0.126
*S* = 1.03
5816 reflections
355 parameters
H-atom parameters constrained
Δρ_max_ = 0.25 e Å^−3^
Δρ_min_ = −0.26 e Å^−3^

### 

Data collection: *SMART* (Bruker, 1997 ▶); cell refinement: *SAINT* (Bruker, 1997 ▶); data reduction: *SAINT*; program(s) used to solve structure: *SHELXS97* (Sheldrick, 2008 ▶); program(s) used to refine structure: *SHELXL97* (Sheldrick, 2008 ▶); molecular graphics: *SHELXTL* (Sheldrick, 2008 ▶); software used to prepare material for publication: *SHELXTL*.

## Supplementary Material

Crystal structure: contains datablock(s) global, I. DOI: 10.1107/S1600536811043509/ng5249sup1.cif

Structure factors: contains datablock(s) I. DOI: 10.1107/S1600536811043509/ng5249Isup2.hkl

Supplementary material file. DOI: 10.1107/S1600536811043509/ng5249Isup3.cml

Additional supplementary materials:  crystallographic information; 3D view; checkCIF report

## Figures and Tables

**Fig. 1.**
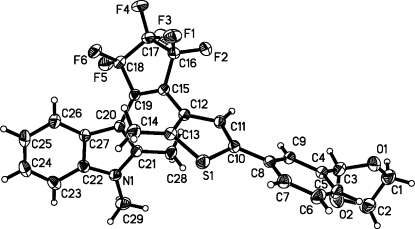
Molecular structure of the title compound with displacement ellipsoids drawn at the 30% probability level. H atoms are represented as small spheres of arbitrary radii.

## supplementary crystallographic information

### Comment

The title compound when dissolved in hexane shows photochromism. Upon irradiation with 297 nm light, the colorless hexane solution turns blue rapidly. The blue compound displays an absorption maximum at 592 nm. Upon irradiation with visible light with wavelength longer than 510 nm, the blue hexane solution reverts to its initial colorless state; a colorless hexane solution of the title compound has an absorption maximum at 278 nm. In a polymethylmethacrylate amorphous film, the title diarylethene also exhibits photochromism similar to that in hexane.

### Experimental

To a tetrahydrofuran solution of 1,2-dimethyl-3-bromoindole (1.12 g, 5 mmol) was added a hexane solution of *n*-butyl lithium 2.5 *M* (2.0 ml, 5 mmol) at 195 K under a nitrogen atmosphere. The mixture was stirred for half an hour. An excess of octafluorocyclopentene (1.5 ml, 10 mmol) was added and stirring was continued for another 2 h at this temperature. The reaction was then quenched by the addition of water. The product, 1,3,3,4,4,5,5-heptafluoro- 2-(1,2-dimethyl-3-indolyl)-3,3,4,4,5,5-hexafluorocyclopent-1-ene (0.76 g, 2.25 mmol), was collected and dried (yield 45.0%). This compound (0.76 g, 2.25 mmol) was reacted with 3-bromo-5-(3-(2,5-dioxolanyl)phenyl)-2-methylthiophene (0.73 g, 2.25 mmol; Irie *et al.*, 2003) in the presence of *n*-butyl lithium 2.5 *M* (0.90 ml, 2.25 mmol) at 195 K under a nitrogen atmosphere. After an hour, the reaction was quenched by the addition of water. The solid product was purified by column chromatography on silica using petroleum ether as the eluant to give the title compound (yield 0.47 g, 0.83 mmol, 37.0%).Crystals suitable for analysis were obtained by slow evaporation of a solution in hexane. Analysis: calculated for C_29_H_13_F_6_NO_2_S:*C* 61.81, H 4.11%; found C 61.84, H 4.20%.

### Refinement

All H atoms were placed in calculated positions with C—H equal 0.93 Å for aromatic and 0.96 Å for CH_3_ groups. They were included in the refinement in the riding model approximation with isotropic displacement parameters set equal to 1.2*U*_eq_(C) and 1.5*U*_eq_(C) of the carrier atom for the aromatic and methyl H atoms, respectively.

### Crystal data

**Table:**

C_29_H_23_F_6_NO_2_S	*F*(000) = 1160
*M**_r_* = 563.54	*D*_x_ = 1.469 Mg m^−^^3^
Monoclinic, *P*2_1_/*c*	Mo *K*α radiation, λ = 0.71073 Å
*a* = 10.7364 (13) Å	Cell parameters from 8530 reflections
*b* = 9.8983 (12) Å	θ = 2.5–28.0°
*c* = 24.020 (3) Å	µ = 0.20 mm^−^^1^
β = 93.151 (1)°	*T* = 296 K
*V* = 2548.8 (5) Å^3^	Block, yellow
*Z* = 4	0.50 × 0.38 × 0.30 mm

### Data collection

**Table:**

Bruker SMART APEX diffractometer	5816 independent reflections
Radiation source: fine-focus sealed tube	4512 reflections with *I* > 2σ(*I*)
graphite	*R*_int_ = 0.024
φ and ω scans	θ_max_ = 27.5°, θ_min_ = 2.6°
Absorption correction: multi-scan (*SADABS*; Sheldrick, 1996)	*h* = −13→13
*T*_min_ = 0.906, *T*_max_ = 0.942	*k* = −12→12
22213 measured reflections	*l* = −31→31

### Refinement

**Table:**

Refinement on *F*^2^	Primary atom site location: structure-invariant direct methods
Least-squares matrix: full	Secondary atom site location: difference Fourier map
*R*[*F*^2^ > 2σ(*F*^2^)] = 0.041	Hydrogen site location: inferred from neighbouring sites
*wR*(*F*^2^) = 0.126	H-atom parameters constrained
*S* = 1.03	*w* = 1/[σ^2^(*F*_o_^2^) + (0.0649*P*)^2^ + 0.789*P*] where *P* = (*F*_o_^2^ + 2*F*_c_^2^)/3
5816 reflections	(Δ/σ)_max_ = 0.011
355 parameters	Δρ_max_ = 0.25 e Å^−^^3^
0 restraints	Δρ_min_ = −0.26 e Å^−^^3^

### Special details

**Table:**

Geometry. All e.s.d.'s (except the e.s.d. in the dihedral angle between two l.s. planes) are estimated using the full covariance matrix. The cell e.s.d.'s are taken into account individually in the estimation of e.s.d.'s in distances, angles and torsion angles; correlations between e.s.d.'s in cell parameters are only used when they are defined by crystal symmetry. An approximate (isotropic) treatment of cell e.s.d.'s is used for estimating e.s.d.'s involving l.s. planes.
Refinement. Refinement of *F*^2^ against ALL reflections. The weighted *R*-factor *wR* and goodness of fit *S* are based on *F*^2^, conventional *R*-factors *R* are based on *F*, with *F* set to zero for negative *F*^2^. The threshold expression of *F*^2^ > σ(*F*^2^) is used only for calculating *R*-factors(gt) *etc*. and is not relevant to the choice of reflections for refinement. *R*-factors based on *F*^2^ are statistically about twice as large as those based on *F*, and *R*- factors based on ALL data will be even larger.

### Fractional atomic coordinates and isotropic or equivalent isotropic displacement parameters (Å^2^)

**Table:**

	*x*	*y*	*z*	*U*_iso_*/*U*_eq_	
S1	0.46926 (4)	0.75926 (5)	0.44072 (2)	0.05075 (14)	
C2	0.5797 (3)	1.4850 (2)	0.26912 (11)	0.0767 (7)	
H2A	0.5468	1.5124	0.2324	0.092*	
H2B	0.6701	1.4859	0.2696	0.092*	
C1	0.5343 (2)	1.5767 (2)	0.31281 (11)	0.0697 (6)	
H1A	0.5936	1.6486	0.3215	0.084*	
H1B	0.4543	1.6162	0.3012	0.084*	
C3	0.4832 (2)	1.3650 (2)	0.33581 (9)	0.0574 (5)	
H3	0.3919	1.3642	0.3311	0.069*	
O1	0.52349 (16)	1.48886 (14)	0.35886 (7)	0.0691 (4)	
O2	0.53560 (19)	1.35553 (16)	0.28268 (7)	0.0788 (5)	
C9	0.44801 (16)	1.14124 (17)	0.38049 (7)	0.0424 (4)	
H9	0.3674	1.1422	0.3642	0.051*	
C8	0.48836 (15)	1.03020 (17)	0.41218 (7)	0.0394 (4)	
C7	0.60943 (17)	1.0324 (2)	0.43654 (8)	0.0477 (4)	
H7	0.6381	0.9596	0.4581	0.057*	
C6	0.68702 (18)	1.1412 (2)	0.42901 (8)	0.0550 (5)	
H6	0.7676	1.1409	0.4454	0.066*	
C4	0.52623 (17)	1.25041 (18)	0.37281 (8)	0.0453 (4)	
C5	0.64630 (18)	1.2503 (2)	0.39749 (8)	0.0523 (5)	
H5	0.6989	1.3235	0.3927	0.063*	
C10	0.40687 (16)	0.91279 (16)	0.41941 (7)	0.0392 (4)	
C13	0.32653 (16)	0.68064 (17)	0.43779 (7)	0.0438 (4)	
C12	0.23425 (15)	0.76930 (16)	0.42070 (7)	0.0372 (3)	
C11	0.28040 (15)	0.90194 (16)	0.41100 (7)	0.0390 (4)	
H11	0.2293	0.9741	0.4000	0.047*	
C14	0.3162 (2)	0.5367 (2)	0.45587 (10)	0.0615 (5)	
H14A	0.2382	0.5235	0.4728	0.092*	
H14B	0.3837	0.5157	0.4824	0.092*	
H14C	0.3201	0.4785	0.4240	0.092*	
C19	0.04988 (15)	0.62631 (16)	0.38624 (6)	0.0351 (3)	
C15	0.10223 (15)	0.72872 (15)	0.41630 (6)	0.0357 (3)	
C18	−0.08018 (16)	0.60133 (17)	0.40400 (7)	0.0412 (4)	
C16	0.00880 (17)	0.79637 (17)	0.45098 (7)	0.0419 (4)	
C17	−0.11661 (17)	0.73266 (19)	0.43227 (8)	0.0467 (4)	
C27	0.09376 (15)	0.40600 (16)	0.33100 (7)	0.0388 (3)	
C21	0.15871 (16)	0.60655 (17)	0.29688 (7)	0.0394 (4)	
C20	0.10126 (15)	0.54940 (16)	0.34138 (6)	0.0367 (3)	
C26	0.05000 (19)	0.29491 (18)	0.35967 (8)	0.0495 (4)	
H26	0.0145	0.3059	0.3938	0.059*	
C22	0.14977 (16)	0.38410 (17)	0.28003 (7)	0.0424 (4)	
C23	0.1598 (2)	0.25562 (19)	0.25678 (9)	0.0539 (5)	
H23	0.1963	0.2429	0.2230	0.065*	
C25	0.0604 (2)	0.16781 (19)	0.33630 (10)	0.0590 (5)	
H25	0.0308	0.0932	0.3551	0.071*	
C24	0.1139 (2)	0.1490 (2)	0.28563 (10)	0.0604 (5)	
H24	0.1186	0.0623	0.2710	0.072*	
C28	0.17998 (19)	0.75138 (18)	0.28411 (8)	0.0500 (4)	
H28A	0.2667	0.7725	0.2910	0.075*	
H28B	0.1309	0.8066	0.3074	0.075*	
H28C	0.1561	0.7686	0.2457	0.075*	
C29	0.2481 (2)	0.5262 (2)	0.20780 (8)	0.0638 (6)	
H29A	0.3058	0.4539	0.2025	0.096*	
H29B	0.2919	0.6108	0.2086	0.096*	
H29C	0.1855	0.5265	0.1777	0.096*	
N1	0.18883 (14)	0.50697 (15)	0.26043 (6)	0.0442 (3)	
F6	−0.08404 (11)	0.49895 (11)	0.44167 (5)	0.0586 (3)	
F5	−0.16255 (10)	0.56709 (13)	0.36180 (5)	0.0624 (3)	
F4	−0.19297 (12)	0.71618 (14)	0.47397 (6)	0.0710 (4)	
F2	0.00594 (12)	0.93212 (11)	0.44437 (6)	0.0672 (3)	
F1	0.03219 (11)	0.77538 (14)	0.50619 (4)	0.0639 (3)	
F3	−0.17684 (12)	0.81256 (14)	0.39387 (6)	0.0727 (4)	

### Atomic displacement parameters (Å^2^)

**Table:**

	*U*^11^	*U*^22^	*U*^33^	*U*^12^	*U*^13^	*U*^23^
S1	0.0373 (2)	0.0444 (3)	0.0708 (3)	0.00047 (18)	0.0052 (2)	0.0026 (2)
C2	0.0806 (16)	0.0674 (15)	0.0844 (16)	−0.0038 (12)	0.0267 (13)	0.0159 (12)
C1	0.0631 (14)	0.0481 (11)	0.0991 (18)	−0.0011 (10)	0.0144 (12)	0.0125 (11)
C3	0.0507 (11)	0.0501 (11)	0.0722 (13)	−0.0103 (9)	0.0109 (10)	0.0030 (9)
O1	0.0832 (11)	0.0417 (7)	0.0850 (11)	−0.0074 (7)	0.0272 (9)	−0.0044 (7)
O2	0.1211 (15)	0.0564 (9)	0.0603 (9)	−0.0133 (9)	0.0182 (9)	0.0006 (7)
C9	0.0356 (8)	0.0440 (9)	0.0476 (9)	−0.0075 (7)	0.0042 (7)	−0.0071 (7)
C8	0.0384 (8)	0.0429 (9)	0.0374 (8)	−0.0070 (7)	0.0063 (6)	−0.0094 (7)
C7	0.0415 (9)	0.0552 (11)	0.0461 (9)	−0.0045 (8)	0.0002 (7)	−0.0056 (8)
C6	0.0385 (9)	0.0708 (13)	0.0552 (11)	−0.0129 (9)	−0.0019 (8)	−0.0087 (10)
C4	0.0434 (9)	0.0431 (9)	0.0502 (10)	−0.0082 (7)	0.0109 (7)	−0.0078 (7)
C5	0.0448 (10)	0.0540 (11)	0.0589 (11)	−0.0199 (8)	0.0092 (8)	−0.0110 (9)
C10	0.0411 (9)	0.0377 (8)	0.0391 (8)	−0.0045 (7)	0.0053 (7)	−0.0053 (6)
C13	0.0425 (9)	0.0388 (9)	0.0508 (10)	−0.0024 (7)	0.0091 (7)	−0.0007 (7)
C12	0.0391 (8)	0.0367 (8)	0.0361 (8)	−0.0040 (6)	0.0060 (6)	−0.0034 (6)
C11	0.0392 (8)	0.0352 (8)	0.0425 (8)	−0.0029 (6)	0.0013 (7)	−0.0012 (6)
C14	0.0535 (12)	0.0451 (11)	0.0870 (15)	0.0018 (9)	0.0132 (11)	0.0143 (10)
C19	0.0383 (8)	0.0342 (8)	0.0330 (7)	−0.0025 (6)	0.0045 (6)	0.0039 (6)
C15	0.0383 (8)	0.0343 (8)	0.0349 (7)	−0.0024 (6)	0.0059 (6)	0.0021 (6)
C18	0.0384 (9)	0.0442 (9)	0.0409 (8)	−0.0072 (7)	0.0021 (7)	0.0051 (7)
C16	0.0494 (10)	0.0380 (8)	0.0391 (8)	−0.0008 (7)	0.0109 (7)	−0.0005 (7)
C17	0.0409 (9)	0.0526 (10)	0.0476 (9)	0.0046 (8)	0.0105 (7)	0.0065 (8)
C27	0.0403 (9)	0.0367 (8)	0.0393 (8)	−0.0028 (7)	−0.0003 (7)	−0.0005 (6)
C21	0.0408 (9)	0.0404 (9)	0.0373 (8)	−0.0059 (7)	0.0040 (7)	−0.0007 (6)
C20	0.0395 (8)	0.0350 (8)	0.0358 (8)	−0.0060 (6)	0.0030 (6)	0.0002 (6)
C26	0.0570 (11)	0.0399 (9)	0.0515 (10)	−0.0055 (8)	0.0033 (8)	0.0063 (8)
C22	0.0433 (9)	0.0405 (9)	0.0431 (9)	−0.0005 (7)	0.0004 (7)	−0.0043 (7)
C23	0.0571 (11)	0.0481 (10)	0.0564 (11)	0.0054 (9)	0.0019 (9)	−0.0123 (8)
C25	0.0664 (13)	0.0358 (9)	0.0739 (13)	−0.0056 (9)	−0.0037 (11)	0.0095 (9)
C24	0.0649 (13)	0.0373 (10)	0.0779 (14)	0.0043 (9)	−0.0067 (11)	−0.0090 (9)
C28	0.0606 (11)	0.0437 (10)	0.0465 (10)	−0.0117 (8)	0.0104 (8)	0.0052 (7)
C29	0.0730 (14)	0.0741 (14)	0.0465 (10)	−0.0085 (11)	0.0246 (10)	−0.0059 (10)
N1	0.0489 (8)	0.0463 (8)	0.0385 (7)	−0.0040 (6)	0.0108 (6)	−0.0025 (6)
F6	0.0633 (7)	0.0500 (6)	0.0646 (7)	−0.0054 (5)	0.0230 (6)	0.0172 (5)
F5	0.0442 (6)	0.0782 (8)	0.0638 (7)	−0.0133 (5)	−0.0051 (5)	−0.0084 (6)
F4	0.0568 (7)	0.0845 (9)	0.0753 (8)	−0.0072 (6)	0.0349 (6)	−0.0066 (7)
F2	0.0781 (8)	0.0367 (6)	0.0900 (9)	0.0017 (5)	0.0336 (7)	−0.0054 (6)
F1	0.0649 (8)	0.0909 (9)	0.0365 (5)	−0.0039 (6)	0.0086 (5)	−0.0064 (5)
F3	0.0695 (8)	0.0693 (8)	0.0777 (8)	0.0233 (7)	−0.0101 (7)	0.0093 (7)

### Geometric parameters (Å, °)

**Table:**

S1—C13	1.7167 (18)	C19—C20	1.453 (2)
S1—C10	1.7269 (18)	C19—C18	1.503 (2)
C2—O2	1.411 (3)	C15—C16	1.497 (2)
C2—C1	1.490 (4)	C18—F5	1.351 (2)
C2—H2A	0.9700	C18—F6	1.3608 (19)
C2—H2B	0.9700	C18—C17	1.527 (3)
C1—O1	1.417 (3)	C16—F1	1.352 (2)
C1—H1A	0.9700	C16—F2	1.353 (2)
C1—H1B	0.9700	C16—C17	1.532 (3)
C3—O1	1.404 (2)	C17—F4	1.339 (2)
C3—O2	1.426 (3)	C17—F3	1.352 (2)
C3—C4	1.498 (3)	C27—C26	1.393 (2)
C3—H3	0.9800	C27—C22	1.410 (2)
C9—C4	1.387 (2)	C27—C20	1.443 (2)
C9—C8	1.392 (2)	C21—N1	1.369 (2)
C9—H9	0.9300	C21—C20	1.384 (2)
C8—C7	1.396 (2)	C21—C28	1.486 (2)
C8—C10	1.471 (2)	C26—C25	1.385 (3)
C7—C6	1.380 (3)	C26—H26	0.9300
C7—H7	0.9300	C22—N1	1.378 (2)
C6—C5	1.376 (3)	C22—C23	1.395 (2)
C6—H6	0.9300	C23—C24	1.369 (3)
C4—C5	1.389 (3)	C23—H23	0.9300
C5—H5	0.9300	C25—C24	1.387 (3)
C10—C11	1.366 (2)	C25—H25	0.9300
C13—C12	1.369 (2)	C24—H24	0.9300
C13—C14	1.496 (2)	C28—H28A	0.9600
C12—C11	1.427 (2)	C28—H28B	0.9600
C12—C15	1.471 (2)	C28—H28C	0.9600
C11—H11	0.9300	C29—N1	1.458 (2)
C14—H14A	0.9600	C29—H29A	0.9600
C14—H14B	0.9600	C29—H29B	0.9600
C14—H14C	0.9600	C29—H29C	0.9600
C19—C15	1.349 (2)		
			
C13—S1—C10	93.21 (8)	C19—C15—C12	127.95 (15)
O2—C2—C1	105.30 (19)	C19—C15—C16	111.21 (14)
O2—C2—H2A	110.7	C12—C15—C16	120.68 (14)
C1—C2—H2A	110.7	F5—C18—F6	105.62 (13)
O2—C2—H2B	110.7	F5—C18—C19	114.11 (14)
C1—C2—H2B	110.7	F6—C18—C19	111.96 (14)
H2A—C2—H2B	108.8	F5—C18—C17	111.96 (15)
O1—C1—C2	102.70 (18)	F6—C18—C17	108.68 (14)
O1—C1—H1A	111.2	C19—C18—C17	104.53 (13)
C2—C1—H1A	111.2	F1—C16—F2	105.63 (14)
O1—C1—H1B	111.2	F1—C16—C15	112.52 (15)
C2—C1—H1B	111.2	F2—C16—C15	112.99 (13)
H1A—C1—H1B	109.1	F1—C16—C17	109.72 (14)
O1—C3—O2	106.39 (15)	F2—C16—C17	111.13 (15)
O1—C3—C4	110.41 (17)	C15—C16—C17	104.93 (14)
O2—C3—C4	110.92 (17)	F4—C17—F3	106.98 (15)
O1—C3—H3	109.7	F4—C17—C18	114.29 (15)
O2—C3—H3	109.7	F3—C17—C18	108.60 (15)
C4—C3—H3	109.7	F4—C17—C16	113.28 (15)
C3—O1—C1	105.36 (17)	F3—C17—C16	109.82 (15)
C2—O2—C3	107.51 (17)	C18—C17—C16	103.79 (14)
C4—C9—C8	121.01 (17)	C26—C27—C22	118.65 (16)
C4—C9—H9	119.5	C26—C27—C20	135.12 (16)
C8—C9—H9	119.5	C22—C27—C20	106.19 (14)
C9—C8—C7	118.14 (16)	N1—C21—C20	109.31 (14)
C9—C8—C10	121.28 (15)	N1—C21—C28	121.21 (15)
C7—C8—C10	120.58 (16)	C20—C21—C28	129.32 (16)
C6—C7—C8	120.79 (19)	C21—C20—C27	106.96 (14)
C6—C7—H7	119.6	C21—C20—C19	124.20 (15)
C8—C7—H7	119.6	C27—C20—C19	128.61 (14)
C7—C6—C5	120.59 (18)	C25—C26—C27	118.68 (18)
C7—C6—H6	119.7	C25—C26—H26	120.7
C5—C6—H6	119.7	C27—C26—H26	120.7
C9—C4—C5	119.80 (18)	N1—C22—C23	129.50 (17)
C9—C4—C3	119.96 (17)	N1—C22—C27	108.26 (14)
C5—C4—C3	120.18 (17)	C23—C22—C27	122.24 (17)
C6—C5—C4	119.67 (17)	C24—C23—C22	117.54 (19)
C6—C5—H5	120.2	C24—C23—H23	121.2
C4—C5—H5	120.2	C22—C23—H23	121.2
C11—C10—C8	129.61 (16)	C26—C25—C24	121.65 (19)
C11—C10—S1	109.93 (12)	C26—C25—H25	119.2
C8—C10—S1	120.45 (13)	C24—C25—H25	119.2
C12—C13—C14	129.32 (17)	C23—C24—C25	121.21 (18)
C12—C13—S1	110.56 (13)	C23—C24—H24	119.4
C14—C13—S1	120.02 (14)	C25—C24—H24	119.4
C13—C12—C11	112.74 (15)	C21—C28—H28A	109.5
C13—C12—C15	121.65 (15)	C21—C28—H28B	109.5
C11—C12—C15	125.54 (15)	H28A—C28—H28B	109.5
C10—C11—C12	113.54 (15)	C21—C28—H28C	109.5
C10—C11—H11	123.2	H28A—C28—H28C	109.5
C12—C11—H11	123.2	H28B—C28—H28C	109.5
C13—C14—H14A	109.5	N1—C29—H29A	109.5
C13—C14—H14B	109.5	N1—C29—H29B	109.5
H14A—C14—H14B	109.5	H29A—C29—H29B	109.5
C13—C14—H14C	109.5	N1—C29—H29C	109.5
H14A—C14—H14C	109.5	H29A—C29—H29C	109.5
H14B—C14—H14C	109.5	H29B—C29—H29C	109.5
C15—C19—C20	128.81 (15)	C21—N1—C22	109.27 (13)
C15—C19—C18	109.75 (14)	C21—N1—C29	126.17 (16)
C20—C19—C18	121.40 (14)	C22—N1—C29	124.52 (15)
			
O2—C2—C1—O1	26.9 (3)	C19—C15—C16—F2	−128.64 (16)
O2—C3—O1—C1	31.8 (2)	C12—C15—C16—F2	55.7 (2)
C4—C3—O1—C1	152.24 (17)	C19—C15—C16—C17	−7.41 (18)
C2—C1—O1—C3	−35.9 (2)	C12—C15—C16—C17	176.91 (14)
C1—C2—O2—C3	−8.2 (3)	F5—C18—C17—F4	88.26 (18)
O1—C3—O2—C2	−14.1 (3)	F6—C18—C17—F4	−28.0 (2)
C4—C3—O2—C2	−134.2 (2)	C19—C18—C17—F4	−147.72 (15)
C4—C9—C8—C7	0.7 (2)	F5—C18—C17—F3	−31.07 (19)
C4—C9—C8—C10	−178.57 (15)	F6—C18—C17—F3	−147.36 (14)
C9—C8—C7—C6	−0.5 (3)	C19—C18—C17—F3	92.94 (16)
C10—C8—C7—C6	178.79 (16)	F5—C18—C17—C16	−147.87 (14)
C8—C7—C6—C5	0.3 (3)	F6—C18—C17—C16	95.84 (15)
C8—C9—C4—C5	−0.7 (3)	C19—C18—C17—C16	−23.86 (16)
C8—C9—C4—C3	176.42 (16)	F1—C16—C17—F4	22.7 (2)
O1—C3—C4—C9	140.83 (17)	F2—C16—C17—F4	−93.70 (18)
O2—C3—C4—C9	−101.5 (2)	C15—C16—C17—F4	143.86 (15)
O1—C3—C4—C5	−42.0 (2)	F1—C16—C17—F3	142.28 (15)
O2—C3—C4—C5	75.6 (2)	F2—C16—C17—F3	25.83 (19)
C7—C6—C5—C4	−0.3 (3)	C15—C16—C17—F3	−96.61 (16)
C9—C4—C5—C6	0.5 (3)	F1—C16—C17—C18	−101.77 (15)
C3—C4—C5—C6	−176.59 (18)	F2—C16—C17—C18	141.78 (14)
C9—C8—C10—C11	−17.9 (3)	C15—C16—C17—C18	19.34 (17)
C7—C8—C10—C11	162.90 (17)	N1—C21—C20—C27	−1.06 (19)
C9—C8—C10—S1	161.20 (13)	C28—C21—C20—C27	174.27 (18)
C7—C8—C10—S1	−18.0 (2)	N1—C21—C20—C19	−176.00 (15)
C13—S1—C10—C11	0.47 (13)	C28—C21—C20—C19	−0.7 (3)
C13—S1—C10—C8	−178.77 (14)	C26—C27—C20—C21	178.3 (2)
C10—S1—C13—C12	0.33 (14)	C22—C27—C20—C21	0.73 (19)
C10—S1—C13—C14	−176.36 (16)	C26—C27—C20—C19	−7.1 (3)
C14—C13—C12—C11	175.27 (19)	C22—C27—C20—C19	175.36 (16)
S1—C13—C12—C11	−1.02 (19)	C15—C19—C20—C21	−47.4 (3)
C14—C13—C12—C15	−1.8 (3)	C18—C19—C20—C21	130.03 (17)
S1—C13—C12—C15	−178.09 (12)	C15—C19—C20—C27	138.83 (19)
C8—C10—C11—C12	178.01 (15)	C18—C19—C20—C27	−43.8 (2)
S1—C10—C11—C12	−1.14 (18)	C22—C27—C26—C25	−1.6 (3)
C13—C12—C11—C10	1.4 (2)	C20—C27—C26—C25	−178.92 (19)
C15—C12—C11—C10	178.36 (15)	C26—C27—C22—N1	−178.17 (16)
C20—C19—C15—C12	−15.4 (3)	C20—C27—C22—N1	−0.14 (19)
C18—C19—C15—C12	166.98 (15)	C26—C27—C22—C23	1.7 (3)
C20—C19—C15—C16	169.35 (16)	C20—C27—C22—C23	179.72 (17)
C18—C19—C15—C16	−8.30 (19)	N1—C22—C23—C24	179.24 (19)
C13—C12—C15—C19	−56.5 (2)	C27—C22—C23—C24	−0.6 (3)
C11—C12—C15—C19	126.83 (19)	C27—C26—C25—C24	0.5 (3)
C13—C12—C15—C16	118.39 (18)	C22—C23—C24—C25	−0.6 (3)
C11—C12—C15—C16	−58.3 (2)	C26—C25—C24—C23	0.6 (3)
C15—C19—C18—F5	143.23 (15)	C20—C21—N1—C22	1.0 (2)
C20—C19—C18—F5	−34.6 (2)	C28—C21—N1—C22	−174.78 (17)
C15—C19—C18—F6	−96.87 (17)	C20—C21—N1—C29	178.59 (18)
C20—C19—C18—F6	85.28 (18)	C28—C21—N1—C29	2.8 (3)
C15—C19—C18—C17	20.60 (18)	C23—C22—N1—C21	179.63 (19)
C20—C19—C18—C17	−157.26 (15)	C27—C22—N1—C21	−0.52 (19)
C19—C15—C16—F1	111.83 (16)	C23—C22—N1—C29	2.0 (3)
C12—C15—C16—F1	−63.85 (19)	C27—C22—N1—C29	−178.16 (18)
